# Patient-reported distress at a cancer center during the COVID-19 pandemic

**DOI:** 10.1038/s41598-023-36025-3

**Published:** 2023-06-13

**Authors:** Manan P. Shah, Sarah W. Rosenthal, Mohana Roy, Ali Raza Khaki, Tina Hernandez-Boussard, Kavitha Ramchandran

**Affiliations:** 1 Department of Medicine, Stanford University, 300 Pasteur Drive, Lane 145, Palo Alto, CA 94304 USA; 2 Department of Biomedical Data Sciences, Stanford University, Palo Alto, USA; 3 Department of Surgery, Stanford University, Palo Alto, USA

**Keywords:** Cancer, Health services, Quality of life

## Abstract

Assessments of health-related quality of life (HRQOL) are conducted by health systems to improve patient-centered care. Studies have shown that the COVID-19 pandemic poses unique stressors for patients with cancer. This study investigates change in self-reported global health scores in patients with cancer before and during the COVID-19 pandemic. In this single-institution retrospective cohort study, patients who completed the Patient-Reported Outcomes Measurement Information System (PROMIS) at a comprehensive cancer center before and during the COVID-19 pandemic were identified. Surveys were analyzed to assess change in the global mental health (GMH) and global physical health (GPH) scores at different time periods (pre-COVID: 3/1/5/2019–3/15/2020, surge1: 6/17/2020–9/7/2020, valley1: 9/8/2020–11/16/2020, surge2: 11/17/2020–3/2/2021, and valley2: 3/3/2021–6/15/2021). A total of 25,192 surveys among 7209 patients were included in the study. Mean GMH score for patients before the COVID-19 pandemic (50.57) was similar to those during various periods during the pandemic: surge1 (48.82), valley1 (48.93), surge2 (48.68), valley2 (49.19). Mean GPH score was significantly higher pre-COVID (42.46) than during surge1 (36.88), valley1 (36.90), surge2 (37.33) and valley2 (37.14). During the pandemic, mean GMH (49.00) and GPH (37.37) scores obtained through in-person were similar to mean GMH (48.53) and GPH (36.94) scores obtained through telehealth. At this comprehensive cancer center, patients with cancer reported stable mental health and deteriorating physical health during the COVID-19 pandemic as indicated by the PROMIS survey. Modality of the survey (in-person versus telehealth) did not affect scores.

## Introduction

Due to the COVID-19 pandemic, patients with chronic illnesses faced new stressors, such as limited interaction with medical teams, decreased access to supportive services, no-visitor policies in healthcare settings, financial hardship, and social isolation^1–4^. During the first several months of the pandemic, the general lack of accurate information and patients’ anxiety about contracting the virus drove poor mental health outcomes^5^. Meanwhile, during the later periods of the pandemic, general distress was noted to improve slightly, but was driven by other factors such as social isolation, adjusting to novel routines, and financial hardship^6–8^.

Numerous studies show that patients with cancer are especially affected by the COVID-19 pandemic. For example, patients with cancer are noted to have worse outcomes after diagnosis with COVID-19, including higher mortality rates than patients without cancer. One study in China noted that when compared with patients without cancer, patients with cancer had a 3.5-fold increased risk of getting admitted to the ICU, requiring mechanical ventilation, or dying due to COVID-19^9,10^. It is likely that anticancer therapies contribute to this increased risk, as patients in this study who received chemotherapy or underwent surgery in the 30 days prior to their COVID-19 infection had increased risk of such severe events when compared with patients with cancer who did not have those treatments. In addition to increasing the risk of severe disease due to COVID-19, systemic anticancer therapy has also been associated with higher rates of COVID-19 complications^11,12^. We hypothesize that poorer outcomes for patients with cancer during the pandemic were due to poorer baseline health, immunocompromised state, and increased average age relative to the general population.

The National Comprehensive Cancer Network (NCCN) recommends patient-reported distress screening in routine cancer care, because clinician perception of subjective symptoms such as fatigue and distress, is often significantly lower than patient-reported ratings^13–15^. Patient-reported outcomes (PROs) are considered the gold-standard for measuring patients’ subjective experience of physical and mental symptoms^16,17^. PROMIS (Patient-Reported Outcomes Measurement Information System), developed by the National Institutes of Health (NIH) Common Fund, implements an item response theory (IRT)-calibrated question bank to measure physical and mental health along a standardized scale that mitigates ceiling and floor effects^18,19^. PROMIS surveys have demonstrated remarkable reliability and validity in assessing patient-reported physical and mental distress in patients without cancer^20–22^.

At Stanford Cancer Institute, the PROMIS (Patient-Reported Outcomes Measurement Information System) survey is administered to all patients with active cancer at intervals of 60 days. These surveys are primarily used to measure patient-centered outcomes that enable providers to evaluate and monitor patients’ GMH (global mental health) and GPH (global physical health). The aim of this study is to report changes in the GPH and GMH of patients with cancer before and during the COVID-19 pandemic. We hypothesize that GMH and GPH for our patient population would decline most from pre-pandemic levels during the initial peak of the pandemic and then improve thereafter.

## Data source

Stanford Cancer Institute is a tertiary referral center for patients with cancer and is a designated comprehensive cancer center. In 2011, an interdisciplinary team developed protocols about PRO (patient-reported outcomes) for the cancer center^23^. The team utilized the Adult Global Health 10 survey (Global- 10 v.1.0/1.1), which consists of 10 questions with responses along a 1–5 scale (1–10 scale for pain) about physical and mental health. The surveys are integrated into routine workflows for patients with cancer as follows: at the patient’s oncology appointment or every two months, whichever occurs less frequently. By May 2013, surveys also became available electronically through the online patient portal prior to clinic appointments. If the survey was not completed electronically prior to the appointment, paper surveys were administered during the clinic visit. By 2015, surveys were routinely administered to all patients at the cancer center.

### Ethics

All methods were carried out in accordance with relevant guidelines and regulations. The need for ethical approval for this study and the need for informed consent was waived by Stanford University. The study was conducted with IRB exemption.

### Study period

For this study, five time periods served as the independent variables: pre-COVID (before the COVID-19 pandemic), two surge periods (surge1 and surge2) during the pandemic, and two valley periods (valley1 and valley2) during the pandemic. The surge and valley periods were determined by the state of California’s county color-tier system that indicated levels of COVID-19 cases and dictated public health policy in the area. The pre-COVID period was set as the year leading up to the implementation of COVID-19-related policies in Santa Clara county (3/1/5/2019–3/15/2020). The surge periods were when Santa Clara County was designated as purple-tier, the highest tier in the system (surge1: 6/17/2020–9/7/2020 and surge2: 11/17/2020–3/2/2021). The valley periods were when the county was downgraded to red tier or lower (valley1: 9/8/2020–11/16/2020) and when the state of California discontinued the color tier system due to declining cases (valley2: 3/3/2021–6/15/2021). There were no surveys between 3/16/2020–6/16/2020 as the surveys were being transitioned to the telehealth patient portal. For each survey, the modality of the visit (in-person or telehealth) was also recorded.

### Patient cohort

**Figure 1:**
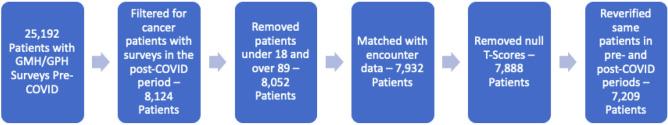
Consort diagram describing cohort identification.

Patients with cancer with at least one completed PROMIS survey in the pre-COVID period and at least one completed PROMIS survey in any of the surge or valley periods were identified. Patients with cancer were defined as patients treated with at least one pre-specified cancer medication (Appendix A). Patients under the age of 18, over the age of 89, and who died within six weeks of being treated were removed from the cohort. Lastly, patients were removed if their generated GMH and GPH scores were null (Fig. 1).

Surveys completed by patients in our cohort during any of the five time periods were collected. Global Mental (GMH) and Physical Health (GPH) scores were generated using the method set forth by Hays et al.14 Survey questions can be found in Appendix A. Each question was answered on a scale from 1 (‘Poor’) to 5 (‘Excellent’).

### Statistical analysis

In each category, scores were totaled and mapped to standardized t-scores according to standardized PROMIS scoring guides. A score of 50 corresponds to the general population and a difference of 3 points suggests clinical significance, as established in prior studies utilizing PROMIS tools for populations with cancer.10 Two-sided t-tests were used to compare GMH and GPH scores.

## Results

Patient demographics (n = 7209) are detailed in Table 1. Mean GMH score for patients with cancer pre-COVID (51) was similar to that of each period during the COVID-19 pandemic (49). Mean GMH score did not vary significantly between surge and valley periods during the pandemic (Table 2). In response to the question, “In general, how would you rate your mental health including your mood and your ability to think? Excellent, Very Good, Good, Fair, Poor,” more patients reported ‘Fair’ or ‘Poor’ mental health pre-COVID (17%) than during the pandemic (14%) (Table 3). Mean GPH score was significantly higher pre-COVID (42) than during the pandemic (37). Mean GPH score did not vary significantly between surge and valley periods during the pandemic (Table 2). During the pandemic, mean GMH and GPH scores obtained in-person were similar to those obtained through telemedicine (Table 4).

**Table 1:** Patient demographics at time of first survey during pre-COVID period.

N = 7209 patients	
Age	
< 45 years	531 (7.37%)
45–59	1601 (22.21%)
60–74	2551 (35.39%)
75 +	1286 (17.84%)
Missing	1801 (24.98%)
Sex	
Male	2000 (27.74%)
Female	3408 (47.27%)
Missing	1801 (24.98%)
Race/Ethnicity	
Asian, Non-Hispanic	1279 (17.74%)
Black, Non-Hispanic	109 (1.51%)
White, Non-Hispanic	3055 (42.38%)
Hispanic	556 (7.71%)
Other/Not reported	409 (5.67%)
Missing	1801 (24.98%)
Language	
Asian	313 (4.34%)
English	4879 (67.68%)
Spanish	143 (1.98%)
Other	72 (1.00%)
Missing	1802 (25.00%)
Cancer type	
Breast	2006 (27.83%)
Cutaneous	224 (3.11%)
Gyn	417 (5.78%)
Lymphoma	544 (7.55%)
Thoracic	524 (7.27%)
Hematology	180 (2.50%)
Urology	725 (10.06%)
GI	692 (9.60%)
Sarcoma	204 (2.83%)
Head and neck	314 (4.36%)
Endocrine	0 (< 1%)
Neuro	0 (< 1%)
Missing	1802 (25.00%)

**Table 2:** Average GMH and GPH scores before and during COVID-19.

Time period	Pre-COVID (N = 7209)	Surge1 (N = 3357)	Valley1 (N = 2877)	Surge2 (N = 4054)	Valley2 (N = 3710)
GMH score	50.57 (9.25)	48.82* (9.07)	48.93* (9.14)	48.68* (8.90)	49.19*^ (9.24)
GPH score	42.46 (9.62)	36.88* (6.72)	36.90* (6.97)	37.33*^ (6.80)	37.14* (6.85)

*Statistically significant difference from the ‘Pre-COVID’ mean at p < 0.001.

^Statistically significant difference from the preceding period’s mean at p < 0.01.

**Table 3:** Response to the question, “In general, how would you rate your mental health including your mood and your ability to think? Excellent, Very Good, Good, Fair, Poor” stratified by COVID-19 time period.

Time period	Pre-COVID (N = 7209)	Surge1 (N = 3357)	Valley1 (N = 2877)	Surge2 (N = 4054)	Valley2 (N = 3710)
% Patients reporting ‘fair or poor mental health, including mood and ability to think’	17.30%	13.64%*	14.36%*	14.38%*	14.50%*

*Statistically significant difference from ‘Pre-COVID’ proportion of patients. None of the post-COVID-19 proportions were statistically different from each other.

**Table 4:** Average GMH and GPH scores by survey modality (in-person versus telehealth) and different periods before and during COVID-19.

Modality	Surge/In-Person (N = 2775)	Surge/Telehealth (N = 3373)
GMH score	49.00 (8.85)	48.53^†^ (9.07)
GPH score	37.37 (6.53)	36.94^ (6.95)

^†^Statistically significant difference from ‘In-Person’ modality during same time period at p < 0.05.

^Statistically significant difference from ‘In-Person’ modality during same time period at p < 0.01.

## Discussion

The COVID-19 pandemic has been a challenging time for all people, with unique stressors for patients with cancer. However, despite the challenging times, in our study surveying patients with cancer through the pandemic, patients reported stable mental health though they did report declining physical health.

Patients with cancer report more fear of contracting COVID-19 and exercise stricter isolation practices than patients without cancer^24^. Before the COVID-19 pandemic, patients with cancer already suffered from higher rates of mental health issues when compared to patients without cancer^25,26^. During the pandemic, worsening mental health has been observed not only in patients with pre-existing mental health conditions, but also in patients with pre-existing chronic medical conditions^24,27,28^. Therefore, we hypothesized that patients with cancer likely suffered from increased mental distress during the pandemic. In many places, the pandemic has also hindered delivery of cancer care, leading to delays in diagnosis/restaging, necessary surgeries, radiation therapies, and other important anticancer treatment^29^. In the setting of COVID-19 infection, patients with cancer often must come off of their anticancer therapy, interrupting their treatment^30^. Reduced access to social events, exercise, and physical therapy might have also led to worsening physical health in patients with chronic illnesses such as cancer^31,32^.

Despite these findings from other studies, patients in our study reported stable mental health during the COVID-19 pandemic, which may be attributable to robust mental health screening and care provided at the cancer center (e.g. social work, palliative care, and psychosocial services)^33^, the rapid transition of medical and supportive services to telehealth^34^, characteristics of the patient population in this relatively affluent region^35^, and/or swift community action in response to the pandemic^36^. One observational study of all patients at a healthcare system reported that GMH scores during the pandemic (47.95) were significantly worse than those before the pandemic (48.47), but these differences were not clinically meaningful^37^. These results echo the relative stability of GMH scores in this study before and during the pandemic. Alternatively, it is also possible that PROMIS is not a sufficiently sensitive tool to detect changes in mental distress in large patient populations due to major events such as the pandemic. Notably, fewer patients reported poor or fair mood during the COVID-19 pandemic than before the pandemic, which is consistent with the relative stability of GMH scores observed in this study.

Notably, mean GPH scores were significantly and meaningfully worse during the COVID-19 pandemic than before the pandemic. While the cancer center was agile in transitioning medical and supportive services to telehealth, it is possible that delays in access to in-person medical care, physical therapy, and exercise programs during the pandemic contributed to worsening physical health. It is also possible that patients with cancer generally have deteriorating GPH over time due to progression of disease. However, in this study, mean GPH score only significantly and meaningfully decreased between the pre-COVID period and the first surge period of the COVID-19 pandemic. Since GPH scores remained stable during the subsequent periods of the pandemic, it is less likely that the decrease in GPH is solely attributable to the effect of time/disease progression in this patient population. These results suggest that improving and maintaining physical health should be a priority for this population during future pandemics requiring lockdowns and isolation. Further exploration is necessary to determine the key drivers of the deteriorating GPH observed in this study.

The results of this study must be interpreted within the context of its limitations as a single-institution, retrospective study utilizing voluntary surveys. It is possible that the patient population served by this cancer center may not be generalizable. An important limitation is that this study does not evaluate changes in GMH and GPH within different cancer subtypes, patients at different stages of disease, or patients from different demographic groups. It is certainly possible that clinical significant changes in certain subsets of patients were masked by the study population at large. Notably, prior research has shown no significant differences in GMH and GPH at baseline among different cancer types, though there were trends towards lower scores among certain racial minorities^23^. Another limitation of this study is the potential for selection bias. For example, it is possible that patients with a language barrier, increased physical or mental distress, or poor technological literacy may not be completing the surveys, and may have been at risk of less access to resources thereby biasing the study population. Since surveys before the COVID-19 pandemic were primarily administered in person, and surveys during the surge and valley period of the pandemic were primarily administered via telehealth, there is also concern for a biasing effect due to lack of control for modality of survey. However, it is reassuring to find that scores obtained via telehealth and in-person during the pandemic were similar, suggesting that modality of survey does not significantly affect PROMIS survey scores.

Studies from other institutions describing GMH and GPH scores before and during the COVID-19 pandemic would provide further context for interpreting the results from this cancer center. Prospective research studies collecting PROMIS scores may also reduce selection bias. It may also prove helpful to analyze responses to other validated mental health screening tools, such as the PHQ-9 and GAD-7, to provide context for the interpretation of PROMIS GMH scores. It would also be interesting to study changes in practice, such as referrals to supportive services, due to changes in PROMIS scores. Such studies would provide deeper understanding of how to utilize PROMIS surveys to assess and guide care during the current and future public health emergencies.

## Conclusions

At this comprehensive cancer center, patients with cancer reported stable mental health and deteriorating physical health during the COVID-19 pandemic. These results suggest that initiatives to improve physical health in patients with cancer may be warranted during public health emergencies such as COVID-19. For example, exercise programs and physical therapy may prevent the deconditioning attributed to the lockdown and remote work. Further research is needed to better understand other causes of physical decline during the pandemic to determine possible targeted interventions. Retrospective reports from other institutions and prospective studies utilizing PROMIS are needed to better understand the role and utility of PROMIS scores in assessing and guiding patient care.

## Supplementary Information

Supplementary Information.

## Data Availability

Database stored in Boussard Lab—available upon request. Contact Manan Shah (mananshah@mednet.ucla.edu) to request data.
