# Supplementary material for: Patient-reported distress at a cancer center during the COVID-19 pandemic
Source: Sci Rep. 2023 Jun 13;13:9581. doi: 10.1038/s41598-023-36025-3 (PMC10262117; doi:10.1038/s41598-023-36025-3)
Supplement: Supplementary file 1 — Supplementary Information. [file 41598_2023_36025_MOESM1_ESM.docx]

**Appendix:**

Appendix A: *Survey Questions*

GMH Questions

- In general, would you say your quality of life is…”
- “In general, how would you rate your mental health including your mood and ability to think?”
- “In general, how would you rate your satisfaction with your social activities and relationships?”
- “In the past 7 days, how often have you been bothered by emotional problems such as feeling anxious, depressed, or irritable?”

GPH Questions

- “In general, how would you rate your physical health?”
- “To what extent are you able to carry out your everyday physical activities such as walking, climbing stairs, carrying groceries, or moving a chair?”
- “In the past 7 days, how would you rate your fatigue?”
- “How would you rate your pain on average?”

Appendix B: *List of anticancer therapies used to identify cohort*

| abemaciclib | ceritinib | everolimus | megestrol | ripretinib |
| --- | --- | --- | --- | --- |
| abiraterone | cetuximab | exemestane | melphalan | rituximab |
| acalabrutinib | chlorambucil | fedratinib | mercaptopurine | romidepsin |
| ado-trastuzumab emtansine | cisplatin | fludarabine phosphate | methotrexate | ruxolitinib |
| afatinib | cladribine | fluorouracil | midostaurin | ruxolitinib phosphate |
| afatinib dimaleate | clofarabine | flutamide | mitomycin | sacituzumab |
| aflibercept | cobimetinib | fostamatinib | necitumumab | siltuximab |
| aldesleukin | crizotinib | fulvestrant | nelarabine | sipuleucel-t |
| alectinib | cyclophosphamide | gefitinib | neratinib | sirolimus |
| alemtuzumab | cytarabine | gemcitabine | nilotinib | sonidegib |
| alpelisib | dabrafenib | gemtuzumab ozogamicin | nilutamide | sorafenib |
| anastrazole | dabrafenib mesylate | gilteritinib | nintedanib | sorafenib tosylate |
| anastrozole | dacarbazine | glasdegib | niraparib | sunitinib |
| apalutamide | dacomitinib | goserelin | nivolumab | sunitinib malate |
| arsenic trioxide | dactinomycin | hydroxyurea | obinutuzumab | talazoparib |
| asparaginase erwinia chrysanthemi | daratumumab | ibritumomab tiuxetan | ofatumumab | tamoxifen citrate |
| atezolizumab | darolutamide | ibrutinib | olaparib | temozolomide |
| avapritinib | dasatinib | idarubicin | omacetaxine mepesuccinate | temsirolimus |
| avelumab | daunorubicin | idelalisib | osimertinib | thalidomide |
| axitinib | decitabine | ifosfamide | osimertinib mesylate | thioguanine |
| azacitidine | degarelix | imatinib | oxaliplatin | thiotepa |
| belinostat | dinutuximab | imatinib mesylate | paclitaxel | tocilizumab |
| bendamustine | docetaxel | ipilimumab | palbociclib | toremifene |
| bevacizumab | doxil | irinotecan | panitumumab | trabectedin |
| bicalutamide | doxorubicin | ivosidenib | panobinostat | trametinib |
| bleomycin sulfate | durvalumab | ixabepilone | pazopanib | trastuzumab |
| blinatumomab | duvelisib | ixazomib citrate | pegaspargase | tucatinib |
| bortezomib | elotuzumab | lanreotide | peginterferon alfa-2b | valrubicin |
| bosutinib | enasidenib | lapatinib | pembrolizumab | vandetanib |
| brentuximab vedotin | encorafenib | lapatinib ditosylate | pemetrexed disodium | vemurafenib |
| brigatinib | enfortumab | larotrectinib | pertuzumab | venetoclax |
| busulfan | entrectinib | lenalidomide | pomalidomide | vinblastine sulfate |
| cabazitaxel | enzalutamide | lenvatinib | ponatinib | vincristine |
| cabozantinib | epirubicin | lenvatinib mesylate | pralatrexate | vincristine sulfate |
| capecitabine | erdafitinib | letrozole | procarbazine | vinorelbine tartrate |
| capmatinib | eribulin mesylate | leucovorin calcium | raloxifene | vismodegib |
| carboplatin | erlotinib | leuprolide | ramucirumab | vorinostat |
| carfilzomib | etoposide | lomustine | regorafenib | ziv-aflibercept |
| carmustine | etoposide phosphate | lorlatinib | ribociclib |  |
